# Estrogen receptor *α* regulates phenotypic switching and proliferation of vascular smooth muscle cells through the NRF1-OMI-mitophagy signaling pathway under simulated microgravity

**DOI:** 10.3389/fphys.2022.1039913

**Published:** 2022-11-10

**Authors:** Min Jiang, Zifan Liu, Junjie Shao, Jingjing Zhou, Haiming Wang, Chao Song, Xin Li, Lin Wang, Qiang Xu, Xiaojuan Liu, Lejian Lin, Ran Zhang

**Affiliations:** ^1^ Department of Pulmonary and Critical Care Medicine, Chinese PLA General Hospital, Beijing, China; ^2^ Department of Cardiovascular Medicine, Chinese PLA General Hospital, Beijing, China; ^3^ Graduate School of Chinese PLA Medical School, Beijing, China; ^4^ Department of Health Services, The First Medical Center of Chinese PLA General Hospital, Beijing, China; ^5^ State Key Laboratory of Kidney Diseases, Chinese PLA General Hospital, Beijing, China

**Keywords:** microgravity, vascular smooth muscle cells, phenotypic switching, proliferation, estrogen receptor *α*, mitophagy, mitochondrial biogenesis, mitochondrial dynamics

## Abstract

Vascular remodeling during microgravity exposure results in postflight cardiovascular deconditioning and orthostatic intolerance in astronauts. To clarify the underlying mechanism, we investigated whether estrogen receptor *α* (ERα)-NRF1-OMI-mitophagy signaling was involved in the dedifferentiation and proliferation of vascular smooth muscle cells (VSMCs) under simulated microgravity. Phenotypic markers, mtDNA copy number and mitochondrial biogenesis, mitochondrial dynamics and mitophagy in rat thoracic artery smooth muscle cells were examined. Four-week hindlimb unweighting (HU) was used to simulate microgravity in rats and 10% serum was used to induce VSMCs dedifferentiation *in vitro*. The effects of ERα-NRF1-OMI signaling on mitophagy, phenotypic switching and proliferation of VSMCs, and cerebrovascular remodeling in HU rats were studied by genetic manipulation and chronic drug intervention. We found that ERα is positively associated with contractile phenotype switching but inversely correlated with synthetic phenotype switching and proliferation of VSMCs both *in vivo* and *in vitro*. During the dedifferentiation process of VSMCs, reduced mtDNA copy number, disturbed mitochondrial biogenesis and respiration, and perturbed fission-fusion-mitophagy signaling were detected, which were reversed by ERα overexpression. Mechanistically, the ERα downstream protein OMI preserved the mitochondrial Parkin level by increasing its protein stability, thereby protecting mitophagy. In line with this, we found that activating ERα signaling by propyl pyrazole triol (PPT) could alleviate the synthetic phenotype switching and proliferation of HU rat cerebral VSMCs by reestablishing fission-fusion-mitophagy hemostasis. The current study clarified a novel mechanism by which inhibited ERα-NRF1-OMI-mitophagy signaling resulted in synthetic phenotype switching and proliferation of VSMCs and cerebrovascular remodeling under simulated microgravity.

## Introduction

Exposure to microgravity during spaceflight results in postflight cardiovascular deconditioning and orthostatic intolerance among astronauts when they return to the earth. Regarding the mechanism, spaceflight is known to impose changes on human vasculature with unknown molecular etiologies. Despite remarkable progress in recent decades, the underlying mechanism remains unelucidated.

Both spaceflight and ground-based studies have revealed adaptive changes in the cardiovascular system under microgravity (Zhang, 2013; Zhang and Hargens, 2018). Accumulating evidence confirms the region-specific structural and functional changes in arteries and microvasculature of different vascular beds during microgravity exposure (Zhang, 2013). Mechanistically, the activation of the renin-angiotensin system and mitochondrial oxidative stress have been implicated in this process (Zhang et al., 2009; Zhang et al., 2014a; Zhang et al., 2014b). We found that mitochondrial oxidative stress and endoplasmic reticulum stress contributed to the phenotypic transition of cerebral vascular smooth muscle cells (VSMCs) through the PERK-eIF2a-ATF4-CHOP pathway in hindlimb unweighting (HU) rats (Zhang et al., 2020a). Mitochondrial dysfunction results in the accumulation of unfolded or misfolded mitochondrial proteins, which triggers the mitochondrial unfolded protein response (UPR^mt^) (Melber and Haynes, 2018). The UPR^mt^ involves the activation of CHOP, estrogen receptor *α* (ERα) and SIRT3 (Papa and Germain, 2014). ERα plays critical roles in regulating mitochondrial function, including bioenergetics, dynamics, and mitophagy (Klinge, 2020). Estrogen/ERα inhibits the proliferation of human VSMCs and prevents their transition from a contractile to a synthetic phenotype (Xia et al., 2020). Regulation of mitochondrial biogenesis involves the coordinated actions of both mitochondrial and nuclear DNA-encoded proteins, including NRF-1, NRF-2, TFAM, and PGC-1α (Klinge, 2017). ERα activation upregulated the mRNA and protein levels of NRF1 and elevated the transcript and protein levels of the protease OMI/HtrA2 in the intermembrane space of mitochondria (IMS) (Münch, 2018). NRF1 can regulate proteasome levels, mitochondrial transcription, and respiration, while OMI is a serine protease that can modulate cellular apoptosis (Münch, 2018). However, further investigation is needed to determine whether the NRF1-OMI signaling pathway is involved in vascular remodeling and phenotype switching and proliferation of VSMCs under simulated microgravity.

In the current study, we investigated whether ERα regulates phenotype switching and proliferation of VSMCs under simulated microgravity. To investigate the underlying mechanism, pharmacological and genetic manipulation of ERα-NRF1-OMI signaling was used to determine whether the NRF1-OMI-mitophagy signaling pathway was involved in this process.

## Materials and methods

All procedures and treatments for animal studies in present study were in accordance with the Guiding Principles for the Care and Use of Animals in the Field of Physiological Sciences and approved by the Animal Institutional Care and Use Committees of Chinese PLA General Hospital.

### Ground-based simulation of microgravity and drug treatment

Hindlimb unweighting (HU) was used to simulate microgravity in male Sprague-Dawley rats weighing 180–200 g as described previously (Zhang et al., 2020a). Briefly, the rat’s tail was hung on a hook at the top of the cage through a chain. Only its forelimbs could contact with the floor of the cage, permitting the rat full access to the entire cage. All rats were caged individually in specific pathogen-free conditions, received standard laboratory chow and water. The room was maintained on a 12:12 h light/dark cycle, 22 ± 1°C. Propyl pyrazole triol (PPT), a selective ERα agonist, was injected subcutaneously from day 3 of modeling at 30 μg/kg/day (Bansal and Chopra, 2018; Butler et al., 2018). 3-MA, an inhibitor of autophagy, was injected intraperitoneally at 25 mg/kg/day. The diagram showing animal modeling is shown in Figure 7A.

### Tissue preparation

After 4-week HU, the rats were anesthetized with 5% isoflurane and euthanized by exsanguination *via* the heart. Cerebral arteries were immediately transferred to ice-cold phosphate buffered saline (PBS), frozen in liquid nitrogen, and stored at −80°C for protein and mRNA abundance analysis. The basilar arteries were rapidly fixed in 4% paraformaldehyde, embedded in paraffin, and sectioned for haematoxylin–eosin and immunohistochemistry staining.

### Haematoxylin-eosin staining

After deparaffinization and rehydration, the nuclei were stained with haematoxylin. The sections were rinsed with PBS, differentiated with 0.3% acid alcohol, and stained with eosin. Finally, the slices were sealed with neutral resins, and images were acquired under a light microscope.

### Immunohistochemistry assay

Antigen retrieval was performed by heating the sections in Tris-EDTA buffer (pH 9.0). Afterwards, the sections were incubated in 0.3% H_2_O_2_ for 10 min, and were then incubated in goat serum for 30 min at 25°C to prevent nonspecific protein-protein interactions. The slices were then incubated with antibodies against α-SMA (1:200, Abcam, Cambridge, MA, United States), OPN (1:200, Abcam, Cambridge, MA, United States) and PCNA (1:200, Abcam, Cambridge, MA, United States) respectively at 4°C for 24 h and then with a biatinylated secondary antibody (ZSGB-BIO, Beijing, China) for 1 h at 25°C, followed by horseradish-peroxidase-labelled avidin (HRP-Avidin) at 25°C for 30 min. Finally, the protein expression of the sections was detected with a diaminobenzidine substrate kit (ZSGB-BIO, Beijing, China) and the nuclei were stained using haematoxylin. The immunoreactivity of α-SMA, OPN, and PCNA in experimental group was quantified relative to that in the CON group using Image-Pro Plus 6.0 (Media Cybernetics, Bethesda, Maryland, United States).

### Cell culture, cell transfections and drug treatment

A7r5 cells (Rat thoracic artery smooth muscle cells) were purchased from Shanghai Zhong Qiao Xin Zhou Biotechnology Co., Ltd. (Item No: ZQ0139). Cells were cultured in DMEM (Hyclone, United States) containing 10% fetal bovine serum (FBS) (Gibco, United States) and 1% penicillin/streptomycin (Gibco, United States). Cells were incubated under 5% CO_2_ at 37°C in humidified incubators. To induce dedifferentiation of VSMCs *in vitro*, A7r5 cells were starved with serum-free medium for 48 h, and were then stimulated with 10% FBS serum as previously described (Zhang et al., 2020b). The cells were harvested at 0, 24, 48, and 72 h for subsequent detection. The A7r5 cells were infected with adenovirus expressing ERα (Ad-ERα), adenovirus expressing OMI (Ad-OMI) or the negative control adenovirus (Ad-NC) in normal culture medium for 48 h. After 48 h or 72 h of siRNA/virus treatment, RNA or protein was extracted for further assays. The siRNA against OMI and the negative control siRNA transfection was performed using Lipofectamine RNA interference (RNAi) max reagent (Thermo Fisher Scientific, United States) according to the manufacturer’s protocol. For inhibitor treatment, 3-MA (10 mM), FCCP (5 μM), MG132 (5 μM) and leupeptin (50 μM) were purchased from MedChemExpress Co., Ltd. (New Jersey, United States).

### Western blot

Total protein of cells was extracted with radio immunoprecipitation assay (RIPA) buffer (50 mM Tris, 250 mM NaCl, pH 7.5, 0.1% SDS, 2 mM dithiothreitol, 0.5% NP-40, 1 mM PMSF, proteases and phosphatases inhibitors) on ice for 20 min. The rat vascular tissues were homogenized and lysed in RIPA buffer on ice for 30 min. After centrifugation at 12,000 g, at 4°C for 10 min, the supernatant was collected and protein concentration was determined by BCA protein assay kit (Pierce, Rockford, IL, United States). Then, proteins were denatured at 100°C for 10 min, separated SDS-PAGE and transferred onto PVDF membranes at 300 mA for 90 min. The membranes were blocked with 5% BSA dissolved in PBS with 0.1% Tween 20 for 1 h at 25°C and then incubated with primary antibodies. Primary antibodies against smooth muscle myosin heavy chain (SM-MHC), α-SMA, calponin, caldesmon, OPN, PCNA, ERα, GAPDH, PGC1α, PGC1β, NRF1, NRF2, Tfam, Plog1, Plog2, Polrmt, β-actin, NDUFB8, SDHB, MTCO1, UQCRC2, ATP5A, FIS1, DRP1, pho-DRP1 (S616), Parkin, PINK1, Mfn1, Mfn2, TOM20, OMI-precursor, OMI-mature, Atg3, Atg7, LC3 I, LC3 II, Beclin1, caspase9 were purchased from were purchased from Abcam (Cambridge, MA, United States), Affinity Biosciences (Affinity Biosciences, OH, United States) and Cell Signaling Technology (Boston, MA, United States). Membranes were washed and incubated with HRP-labelled secondary antibodies for 1 h at 25°C. Membranes were developed using ECL, and ImageJ software (NIH) was used for quantifying band intensities.

### Quantitative polymerase chain reaction

Total RNA of cells and tissues were extracted with TRNzol Reagent (Invitrogen, Carlsbad, CA, United States). 500 ng RNA was reversed transcribed with PrimeScript RT reagent Kit (Takara, kyoto, Japan) following the manufacturer’s instructions. Quantitative Real-time PCR was performed with The SYBR Premix Ex Taq II Kit (Takara, kyoto, Japan) and CFX96 Real-Time PCR Detection System (Bio-Rad). *Gapdh* was the internal control for quantifying mRNA levels. All primers used in this study is shown in Table 1.

**TABLE 1:** Primers used for real-time quantitative polymerase chain reaction.

Target genes	Sequences	
*Esr1*	F	5′-CCC​ACT​CAA​CAG​CGT​GTC​TC-3′
	R	5′-CGT​CGA​TTA​TCT​GAA​TTT​GGC​CT-3′
*Myh11*	F	5′-ATG​AGG​TGG​TCG​TGG​AGT​TG-3′
	R	5′-GCC​TGA​GAA​GTA​TCG​CTC​CC-3′
*Spp1*	F	5′-CTC​CAT​TGA​CTC​GAA​CGA​CTC-3′
	R	5′-CAG​GTC​TGC​GAA​ACT​TCT​TAG​AT-3′
*Pgc1α*	F	5′-AAC​TGG​TGT​CGT​GGA​GTC​GGC-3′
	R	5′-GGC​CTG​GCT​GGA​CAG​AGT​TG-3′
*Pgc1β*	F	5ʹ-TAC​CAG​AGC​CAA​GAA​CGC​TG-3′
	R	5ʹ-CGC​AGT​TGG​CTG​TTG​ATC​TG-3′
*Nrf1*	F	5ʹ-AAC​TGG​TGT​CGT​GGA​GTC​GGC-3’
	R	5ʹ-GGC​CTG​GCT​GGA​CAG​AGT​TG-3’
*Nrf2*	F	5ʹ-TAC​CAG​AGC​CAA​GAA​CGC​TG-3′
	R	5ʹ-CGC​AGT​TGG​CTG​TTG​ATC​TG-3′
*Tfam*	F	5ʹ-GCA​CCG​TCA​AGG​CTG​AGA​AC-3ʹ
	R	5ʹ-TGG​TGA​AGA​CGC​CAG​TGG​A-3ʹ
*Plog1*	F	5ʹ-GCA​CCG​TCA​AGG​CTG​AGA​AC-3ʹ
	R	5ʹ-TGG​TGA​AGA​CGC​CAG​TGG​A-3ʹ
*Plog2*	F	5ʹ-CCG​CTC​GAG​GAG​CTT​CCA​GCT​GAG​CAC​TGG​G-3′
	R	5ʹ-CGA​CGC​GTT​ATT​GCG​CCC​CCA​TCA​GCA​CT-3′
*Polrmt*	F	5ʹ-GCA​CCG​TCA​AGG​CTG​AGA​AC-3ʹ
	R	5ʹ-TGG​TGA​AGA​CGC​CAG​TGG​A-3ʹ
*Gapdh*	F	5ʹ-GCA​CCG​TCA​AGG​CTG​AGA​AC-3ʹ
	R	5ʹ-TGG​TGA​AGA​CGC​CAG​TGG​A-3ʹ

### Ubiquitylation assays

For Parkin ubiquitylation assay, OMI and Parkin vectors were transfected into HEK293T cells using Lipofectamine 3000 (Invitrogen, CA, United States). After 24 h, cells were washed, centrifuged and lysed in lysis buffer (50 mM Tris-HCl, pH 8.0, 150 mM NaCl, 1% Triton X-100, 5 mM EDTA, 0.1% SDS, 0.5% sodium deoxycholate). Cytoplasmic proteins were obtained by centrifugation of lysate and incubated with anti-Myc antibodies for 3 h, followed by incubated with Protein A/G agarose beads at 4°C for 2 h. Then washed the beads 4 times with lysis buffer. The proteins were denatured and detected by western blot analysis.

### Mitochondria isolation

Cells were harvested and suspended in ice-cold MSE buffer [220 mM mannitol, 70 mM sucrose, 5 mM 3-(N-morpholino) propanesulfonic acid, 2 mM ethyleneglycol-bis-(b-aminoethyl ether)-N, N9-tetraacetic acid, pH 7.2, with KOH], homogenized using an ultrasonic cell disrupter system. Unbroken cells and nuclei were precipitated by centrifugated two times at 600 g, 4°C for 5 min. To obtain sketchy mitochondrial and cytosolic fraction, the supernatant was transferred into a new tube and centrifugated at 8,500 g, 4°C for 10 min. Then resuspended the precipitate in 100 μl MSE buffer with phosphatase inhibitors and protease. The mixture was centrifuged at 8,500 g, 4°C for 10 min, and resuspended the precipitate in 50 μl MSE with phosphatase inhibitors and protease.

### Mitochondrial respiration assay

Oxygen consumption rates was used for testing mitochondrial respiration. In detail, the Clark oxygen electrode (500 μl reaction chamber) was water bathed at 37°C. The chamber was filled with fresh respiration buffer until baseline no longer fluctuates. 500 μg isolated mitochondria were added to the chamber, and wait until the oxygen consumption rate was stabilized. The rate of oxygen consumption was lower (state IV) after 5 mM succinate was added. Then added 5 mM ADP to stimulate a high level of oxygen consumption rate (state III). After the added ADP was used up, the oxygen consumption rate switched back from state III rate to the IV rate. Respiratory control ratio (RCR) is the ratio of oxygen intake between states III and IV.

### mtDNA copy number analysis

Total DNA was extracted from cells and tissue following a standard phenol-chloroform protocol. RT-qPCR was used to determine mitochondrial DNA (mtDNA) copy numbers relative to the nuclear DNA content. Mitochondrial gene, ND4, and nuclear gene, 18S, were amplified in the same PCR reaction following a two-step thermal profile: denatured at 95°C for 10 min, followed by 40 cycles of 10 s at 95°C, 60°C for 30 s on the QIAquant96 5plex (QIAGEN). Equation 2^−ΔΔCt^ was used for calculating mitochondrial copy number. ΔCt = Ct (ND4) − Ct (18S).

### Confocal microscopy

Cells grown on confocal dish were fixed with 4% paraformaldehyde for 15 min at room temperature, washed three times with PBS. Then samples were blocked with 1% BSA and permeated by 0.1% Triton X-100 for 1 h at 25°C. After washed three times with PBS, the samples were incubated with primary antibodies against TOMM20 protein (Abcam, Cambridge, MA, United States) at 4 °C overnight. After washing 3 times with PBS, cells were then incubated with secondary antibodies protected from light for 1 h at 25°C. The samples were counterstained with DAPI (1/1000) in PBS. Images were acquired using a confocal microscope (LSM 710, Zeiss).

### Oxygen consumption rate assay

OCR was studied using a Seahorse XFe96 Extracellular Flux Analyzer (Agilent, Santa Clara, CA, United States) following the manufacturer’s protocol. A7r5 cells were seeded in 96-well plate (Agilent, Santa Clara, CA, United States) according to the manufacturer’s instructions and cultured 24 h before measuring OCR. Changes in oxygen consumption were measured following treatment with 5 μM mitochondrial inhibitors oligomycin, 2 μM FCCP, and 1 µM antimycin A. Data were analyzed with the Wave 2.6.2 software package (Agilent, Santa Clara, CA, United States).

### EdU incorporation assay

The 5-ethynyl-2-deoxyuridine (EdU) incorporation assay was used to assess proliferation of A7r5 cells. EdU (BeyoClick, Shanghai, China) was diluted in medium at 50 μmol/L, and the cells were incubated in the medium for 2 h. After removing the medium, the cells were fixed, washed with 2 mg/ml glycine, followed by treating with TrionX-100 and reaction buffer. After stained with DAPI, the cells wereexamined under a fluorescent microscope. Five random microscopic fields in each group were used to determine the proliferation rate, which is expressed as percentage of EdU positive cells.

### Statistical analysis

All quantitative data are presented as mean ± SEM. The two groups were compared using student *t* test for statistical evaluation. One-way analysis of variance (ANOVA) was performed for more than two groups for statistical evaluation. All statistical analyses were performed with GraphPad prism 8.0. Differences were considered significant at *, ^#^ or ^△^ *p* < 0.05, **, ^##^ or ^△△^ *p* < 0.01, and ***, ^###^ or ^△△△^ *p* < 0.001.

## Results

### Estrogen receptor *α* is associated with phenotypic switching of vascular smooth muscle cells *in vitro* and *in vivo*

**FIGURE 1:**
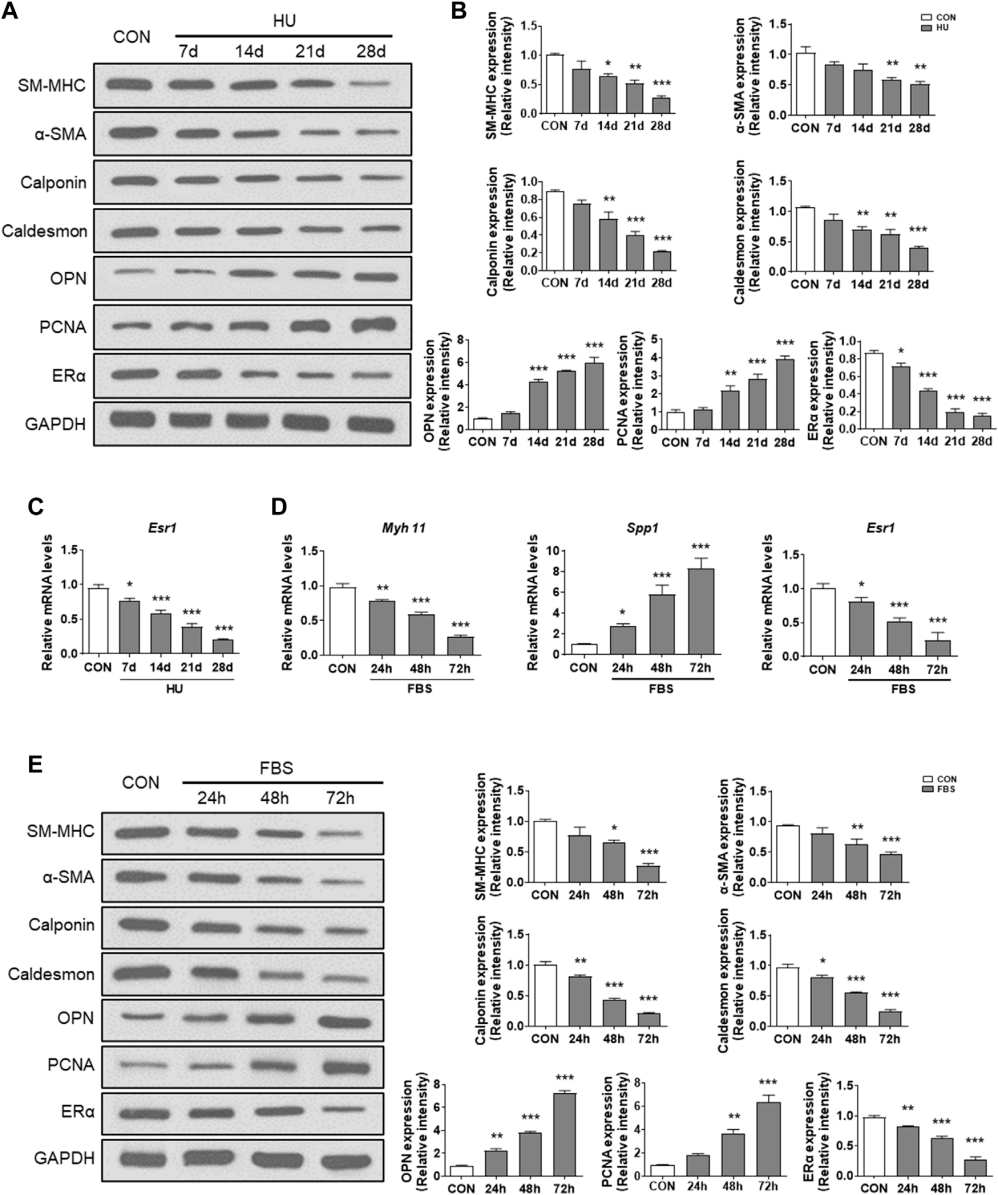
ERα is associated with phenotype switching of VSMCs *in vitro* and *in vivo*. **(A,B)** Western blot analysis of SM-MHC, α-SMA, calponin, caldesmon, OPN, PCNA and ERα protein levels in rat cerebrovascular VSMCs under HU or control **(A)**, and quantification of the protein levels compared to GAPDH **(B)**. **(C,D)**, Western blot analysis of SM-MHC, α-SMA, calponin, caldesmon, OPN, PCNA and ERα protein levels in A7r5 cells treated with FBS **(C)**, and quantification of the protein levels compared to GAPDH **(D)**. **(E)** The mRNA expression levels of *Esr1* in rat cerebrovascular VSMCs under HU or control. **(F)** The mRNA expression levels of *Myh11*, *Spp1*, *Esr1* in A7r5 cells treated with FBS for different hours. CON, control; HU, hindlimb unweighting; Values are the mean ± SEM (*n* = 6 mice or 3 independent cell isolations per group). *p < 0.05, **p < 0.01 and ***p < 0.001 vs. control.

To investigate whether ERα is involved in synthetic phenotype switching of VSMCs under simulated microgravity, we examined the protein/mRNA abundance of phenotypic markers and ERα in HU rat cerebral VSMCs. As shown in Figure 1A,B, compared with controls, the protein abundance of SM-MHC, α-SMA, calponin, caldesmon and ERα gradually decreased, while the protein abundance of OPN and PCNA gradually increased in the HU rat cerebral VSMCs. At the transcriptional level, the mRNA level of *Esr1* also gradually decreased during HU (Figure 1C). *In vitro*, FBS induces synthetic transition of VSMCs (Chen et al., 2011; Zhang et al., 2020b). At the transcriptional level, the mRNA levels of Myh11 and Esr1 gradually decreased, while the mRNA level of Spp1 gradually increased (Figure 1D). In FBS-treated VSMCs, the expression of SM-MHC, α-SMA, calponin, caldesmon and ERα gradually decreased, while the protein levels of OPN and PCNA gradually increased in a timedependent manner (Figures 1E,F). These data suggest that ERα is positively associated with contractile phenotype switching but inversely correlated with synthetic phenotype switching and proliferation of VSMCs, both in *in vivo* HU rat cerebral arteries and *in vitro* VSMCs incubated with FBS.

### Estrogen receptor *α* increases mtDNA copy number and mitochondrial biogenesis

**FIGURE 2:**
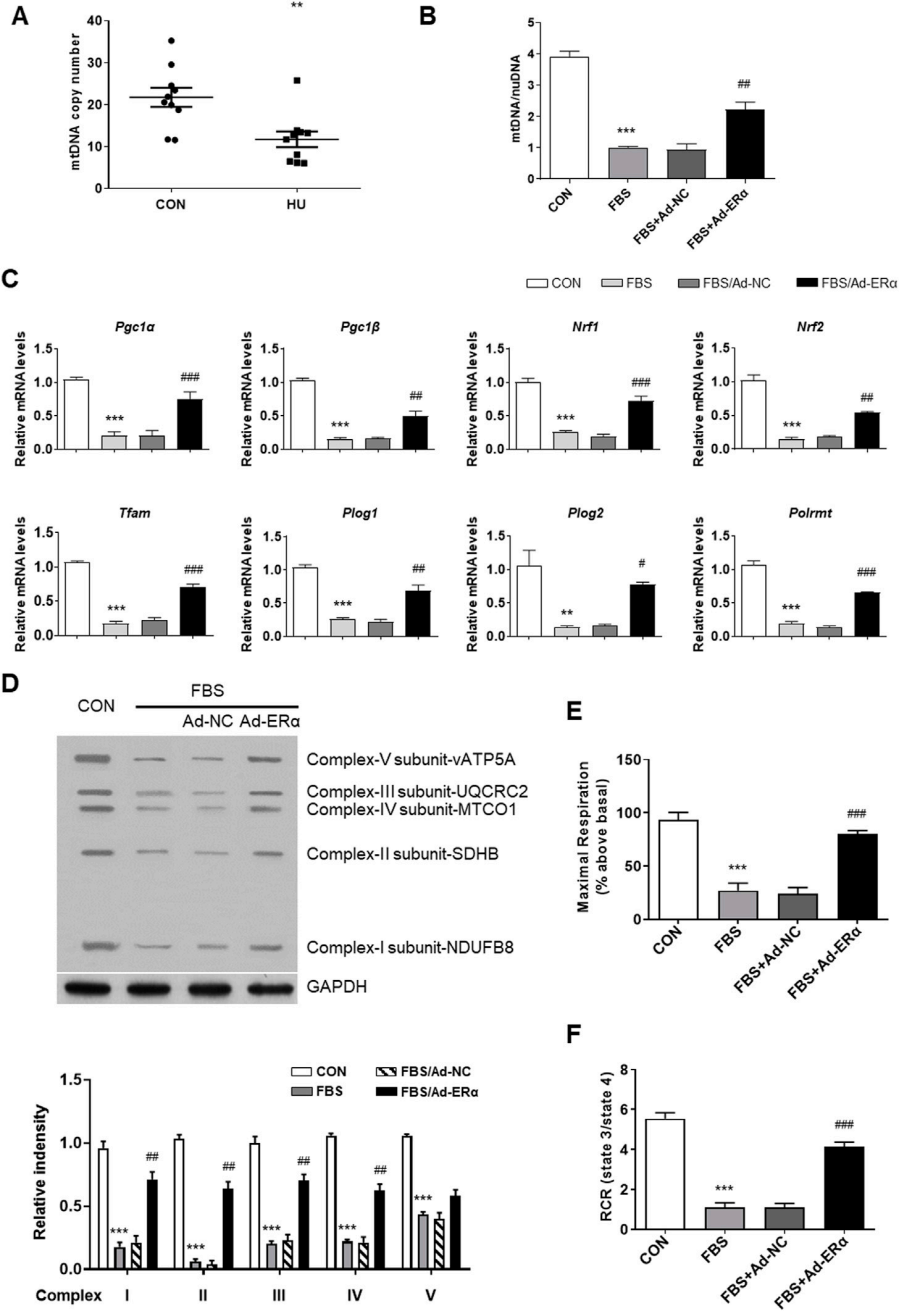
ERα increases mtDNA copy number and mitochondrial biogenesis. **(A)** The mtDNA copy number in cerebral VSMCs (*n* = 10 per group). **(B–F)** The mtDNA copy number **(B)**, the mRNA expression levels of *Pgc1α, Pgc1β, Nrf1, Nrf2, Tfam, Plog1, Plog2*, and *Polrmt* **(C)**, representative subunits of the electron transport chain **(D)**, the maximal cellular respiration **(E)** and mitochondrial respiratory reserve capacity **(F)** in A7r5 cells treated with FBS after transfected with Ad-ERα or Ad-NC (*n* = 3 per group). The A7r5 cells mentioned above were incubated with FBS for 72 h after transfected with adenoviruses expressing ERα or noncoding control for 48 h. *p < 0.05, **p < 0.01 and ***p < 0.001 vs. control; ^#^ *p* < 0.05, ^##^ *p* < 0.01 and ^###^ *p* < 0.001 vs. FBS.

Regulation of mitochondrial biogenesis involves the coordinated actions of both mtDNA and nuclear-encoded gene products, including NRF-1, NRF-2, TFAM, and PGC-1α (Klinge, 2017). Previous studies have shown that ERα regulates mitochondrial function, dynamics, and turnover in pancreatic β-cells and skeletal muscle (Ribas et al., 2016; Zhou et al., 2018). Here, we assessed the effects of ERα on mitochondrial biology in VSMCs. Compared with controls, HU induced a significant reduction in mtDNA copy number in cerebral VSMCs (Figure 2A). Moreover, FBS-treated VSMCs showed a reduced mtDNA number, which was reversed by ERα overexpression (Figure 2B). The above data were further validated by the decreased expression of *Ppargc1α* and genes encoding proteins of the tricarboxylic acid cycle and electron transport chain (ETC), including *Ppargc1β*, *Nrf1*, *Nrf2*, *Tfam*, *Plog1*, *Plog2 and Polrmt,* in FBS-treated VSMCs, which was partially restored by ERα overexpression (Figure 2C). The protein abundance of these genes was also decreased and reversed by ERα overexpression (Supplementary Figure S1). *Polg1* and *Polg2* encode the catalytic and accessory subunits of polymerase γ, respectively, and the latter enhances DNA binding and promotes processive DNA synthesis. ERα can induce *Plog1* expression by binding to the Plog1 promoter (Zhou et al., 2020). Our data suggest that ERα regulates mtDNA replication and transcription in VSMCs. Furthermore, we also detected reduced protein abundance of representative subunits of the electron transport chain, as well as significantly attenuated maximal cellular respiration and mitochondrial respiratory reserve capacity in serum-treated VSMCs, which were all alleviated by ERα overexpression (Figures 2D–F). In conclusion, these findings suggested that ERα controls mtDNA copy number, biogenesis, and respiration of mitochondria during dedifferentiation of VSMCs.

### Estrogen receptor *α* regulates mitochondrial dynamics and mitophagy signaling

**FIGURE 3:**
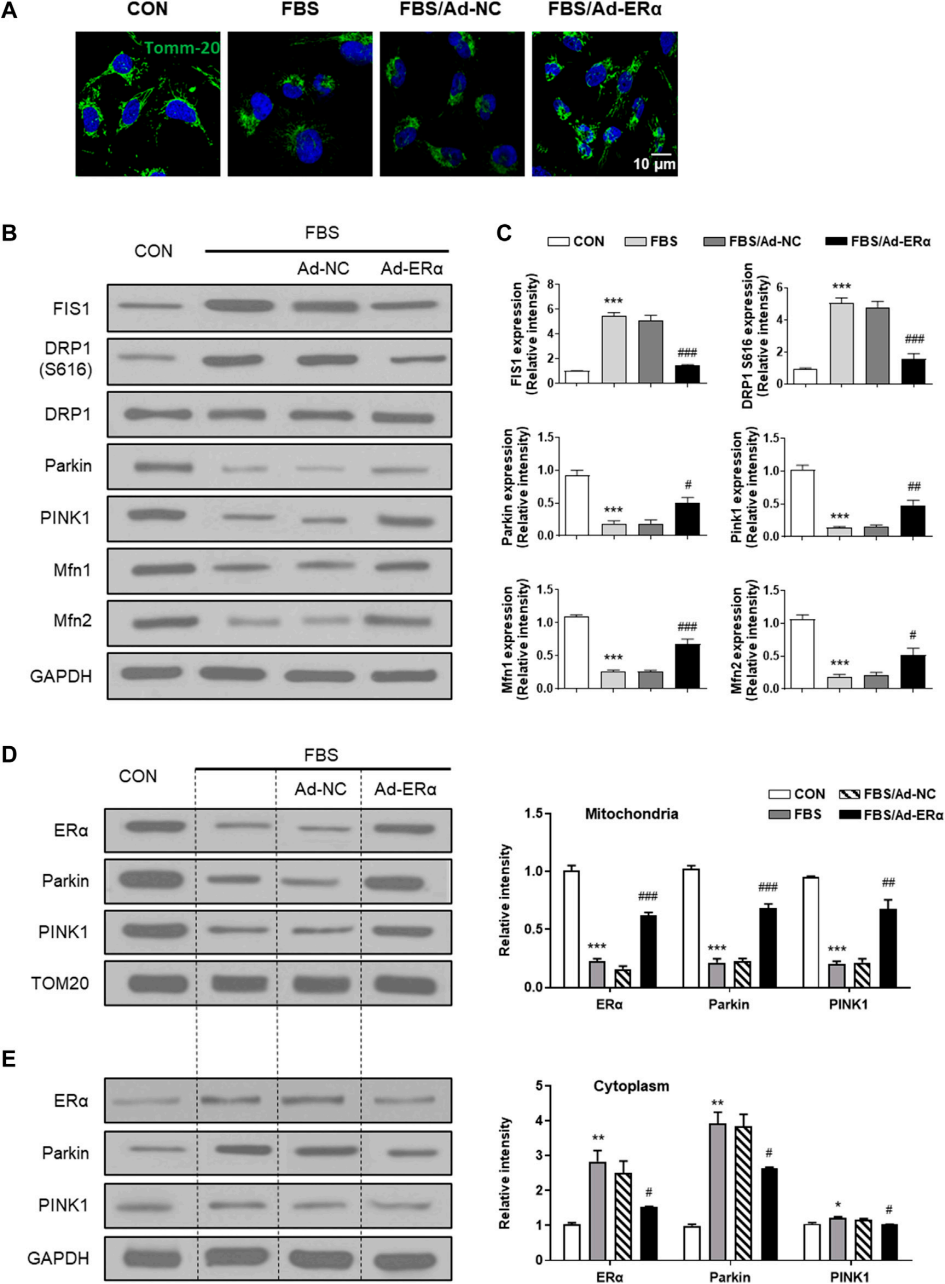
ERα regulates mitochondrial dynamics and mitophagy signaling. **(A)** Representative images of immunofluorescence staining for TOMM20 (green) and DAPI (blue) in A7r5 cells. **(B,C)** Western blot analysis **(B)** and quantification compared to GAPDH **(C)** of FIS1, DRP1 S616, DRP1, Parkin, PINK1, Mfn1 and Mfn2 protein levels in A7r5 cells. **(D,E)** Left, western blot analysis of ERα, Parkin and PINK1 protein levels in mitochondria **(D)** and cytoplasm **(E)** of A7r5 cells, respectively. The A7r5 cells mentioned above were incubated with FBS for 72 h after transfected with adenoviruses expressing ERα or noncoding control for 48 h. *p < 0.05, **p < 0.01 and ***p < 0.001 vs. control; ^#^ *p* < 0.05, ^##^ *p* < 0.01 and ^###^ *p* < 0.001 vs. FBS.

mtDNA replication is closely linked with mitochondrial dynamics and turnover; therefore, we investigated mitochondrial fusion-fission and mitophagy signaling. As shown in Figure 3A, treatment of VSMCs with FBS induced mitochondrial fission, which was significantly inhibited by ERα overexpression. The outer mitochondrial membrane docking protein mitochondrial fission 1 protein (FIS1) and phosphorylation of the mitochondrial fission regulator dynamin-related protein 1 (Drp1) at its activation site Ser616 (DRP S616) were significantly upregulated in FBS-treated VSMCs, and ERα overexpression significantly inhibited them and blocked mitochondrial fission (Figures 3B,C). In addition, mitochondrial fusion proteins (Mfn1 and Mfn2) were decreased in FBS-treated VSMCs, which was also reversed by ERα overexpression. This is consistent with our previous study, which showed an elongated and hyperfused mitochondrial morphology in synthetic VSMCs of cerebral arteries of HU rats (Liu et al., 2021).

In parallel with the diminished fission-fusion dynamics, mitophagy signaling of parkin and pink was attenuated in FBS-treated cells, which was restored by ERα overexpression (Figure 3B). As the accumulation of PINK1 in the OMM is critical for mitophagy initiation, we performed mitochondrial isolation studies. As shown in Figures 3D,E, in mitochondria, the treatment of VSMCs with FBS significantly decreased the expression of ERα, Parkin and PINK1, while in the cytosol, FBS significantly increased the expression of ERα and Parkin, with PINK1 being unchanged. These data suggested that FBS-induced ERα downregulation causes a reduction in Park family protein levels and implicates increased PINK1/Parkin protein turnover as a potential mechanism for inhibited mitophagy signaling. Together, these results support the notion that ERα is critical for mitochondrial health surveillance and mitophagy signaling.

### ERα-NRF1-OMI regulates parkin abundance

**FIGURE 4:**
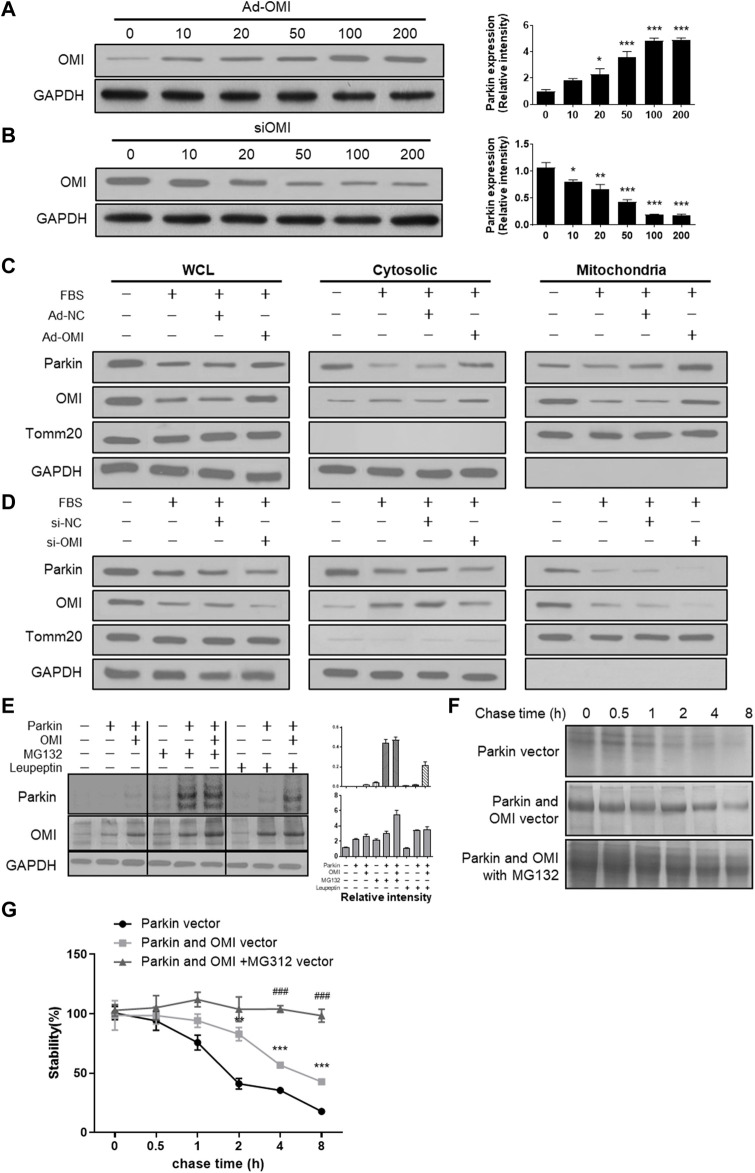
ERα-NRF1-OMI involved in regulating parkin expression. **(A,B)** Western blot analysis of OMI in A7r5 cells transfected with different OMI of adenoviruses **(A)** or siRNA **(B)** expressing OMI. **(C,D)** Western blot analysis of Parkin and OMI protein levels in total cell lysis (left), cytoplasm (middle) and mitochondria (right) of A7r5 cells treated with FBS, transfected with adenoviruses expressing OMI or noncoding control **(C)** and with adenoviruses expressing OMI or noncoding control **(D)**. **(E)** Western blot analysis of parkin and OMI protein levels in HEK293 cells. The cells were transfected with Parkin and OMI vector, and treated with MG132 and/or leupeptin. **(F,G)** Expression of OMI promoted Parkin stability *via* the proteasomal pathway. HEK293 Cells were transfected with Parkin alone [**(F)**; top panel], Parkin and OMI [**(F)**; middle panel], or Parkin and OMI with MG132 treatment [**(F)**; bottom panel], and levels of Parkin were detected by western blot analysis. The representative bolt **(F)** and quantitation of 3 independent experiments are shown **(G)**. *p < 0.05, **p < 0.01 and ***p < 0.001 vs. control.

Activation of the ERα-NRF1-OMI signaling pathway, that is, the UPR^mt^-ERα axis, augments the protein quality control system to prevent the import and accumulation of defective proteins in the IMS. ERα activation upregulated mRNA and protein levels of NRF1 and elevated transcript and protein levels of the protease OMI/HtrA2 in the intermembrane space of mitochondria (IMS) (Münch, 2018). Previous studies suggested that dysfunctional OMI was associated with impaired autophagy (Li et al., 2010), while its roles in regulating mitophagy are unknown. Adenovirus and siRNA of OMI were established to overexpress and downregulate OMI (Figures 4A,B). In FBS-treated VSMCs, OMI overexpression increased the level of parkin in mitochondria but not in the cytosol (Figure 4C). However, OMI knockdown significantly inhibited the expression of parkin in the cytosol and mitochondria, with a more pronounced trend in mitochondria (Figure 4D). As shown in Figure 4E, OMI overexpression in VSMCs increased the steady-state level of Parkin compared with control transfectants, which was remarkably boosted by the proteasome inhibitor MG132 and the protease inhibitor leupeptin. Pulse chase analysis showed that Parkin stability was remarkably augmented by OMI expression, especially in the presence of MG132 (Figures 4F,G). Therefore, these results suggest that OMI inhibits Parkin degradation primarily *via* the ubiquitin‒proteasome system (UPS).

### ERα-NRF1-OMI regulates the macroautophagy and mitophagy flux

**FIGURE 5:**
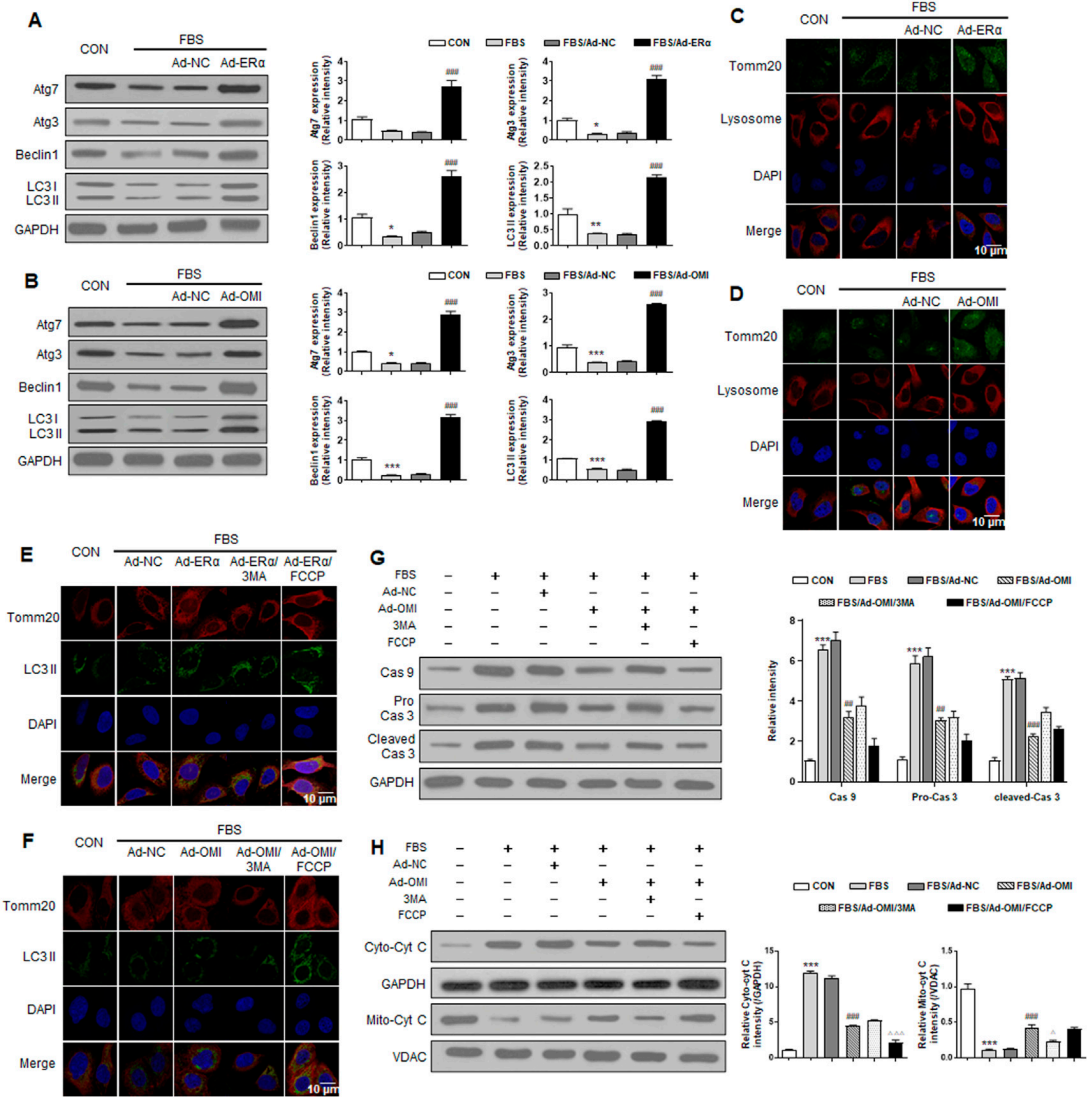
ERα-NRF1-OMI regulates macroautophagy and mitophagy flux. **(A–C)** Western blot analysis of Atg7, Atg3, beclin1, LC3 I and LC3 II **(A)**, the co-staining of TOMM20, lysosome and DAPI **(B)** and the co-staining of TOMM20, LC3 II and DAPI **(C)** in A7r5 cells treated with FBS, transfected with adenoviruses expressing ERα or noncoding control. **(D-H)** Western blot analysis of Atg7, Atg3, beclin1, LC3 I and LC3 II **(D)**, the co-staining of TOMM20, lysosome and DAPI **(E)**, the co-staining of TOMM20, LC3 II and DAPI **(F)**, the expression level of caspase9, caspase3 and cleaved caspase3 **(G)** and cytosolic cyt-c and mitochondrial cyt-c **(H)** in A7r5 cells treated with FBS, transfected with adenoviruses expressing OMI or noncoding control. 3MA and FCCP were used as mitophagy inhibitor and activator respectively. *p < 0.05, **p < 0.01 and ***p < 0.001 vs. control, ^##^ *p* < 0.01 and ^###^ *p* < 0.001 vs. FBS, ^Δ^ *p* < 0.05 and ^ΔΔΔ^ *p* < 0.001 vs. FBS + Ad-OMI.

To elucidate whether ERα-NRF1-OMI regulates mitophagy in A7r5 cells, we evaluated markers of macroautophagy required for mitochondrial turnover by lysosomes, including Beclin, Atg5, Atg7, Atg12, and LC3 processing. The serum significantly inhibited the expression of these markers and the ratio of LC3 II/LC3 I (Figure 5A,B). Immunofluorescence analysis revealed that serum inhibited the overlap between lysosomes and mitochondria in VSMCs (Figures 5C,D). The above alterations were reversed by ERα and OMI overexpression (Figures 5A–D). The mitophagy activator (FCCP) and inhibitor (3-MA) were used to verify whether the repressed mitophagy was associated with defective mitochondrial accumulation and apoptosis activation. Compared with the control, FBS reduced LC3 II and mitochondrial colocalization in VSMCs, which was also protected by ERα and OMI overexpression (Figures 5E,F). FBS upregulated the expression of caspase9/3 and induced cyt-c diffusion into the cytoplasm, which was impeded by ERα and OMI overexpression (Figures 5G–H). Mitophagy inhibition caused cyt-c release and upregulated caspase9/3 expression, while mitophagy activation under ERα and OMI overexpression further inhibited these changes (Figures 5G,H). In conclusion, these data suggest that the treatment of VSMCs with FBS prevents mitophagy from clearing dysfunctional mitochondria and results in increased apoptosis, while the ERα-NRF1-OMI signaling pathway can preserve mitophagy and limit cyt-c leakage.

### ERα-NRF1-OMI regulates proliferation of vascular smooth muscle cells *via* mitophagy

**FIGURE 6:**
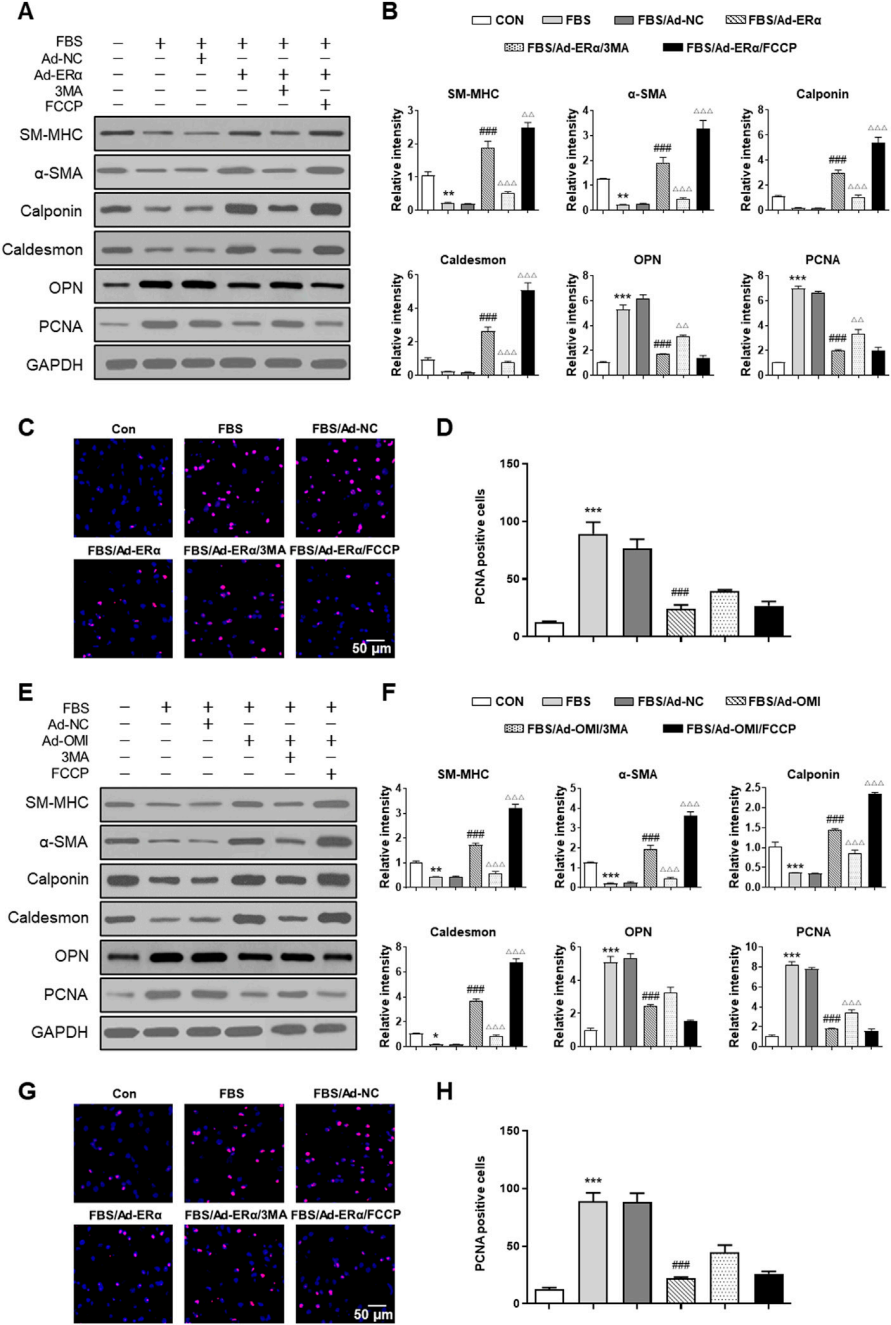
ERα-NRF1-OMI regulates proliferation of VSMCs *via* mitophagy. **(A–D)**, Western blot analysis of SM-MHC, α-SMA, calponin, caldesmon, OPN and PCNA protein **(A,B)** and EdU incorporation assay **(C,D)** in A7r5 cells treated with or without FBS and transfected with Adenoviruses expressing ERα. **(E–H)** Western blot analysis of SM-MHC, α-SMA, calponin, caldesmon, OPN and PCNA protein **(E,F)** and EdU incorporation assay **(G,H)** in A7r5 cells treated with or without FBS and transfected with Adenoviruses expressing OMI. 3MA and FCCP were used as mitophagy inhibitor and activator, respectively. *p < 0.05, **p < 0.01 and ***p < 0.001 vs. control, ^###^ *p* < 0.001 vs. FBS, ^Δ^ *p* < 0.05, ^ΔΔ^ *p* < 0.01 and ^ΔΔΔ^ *p* < 0.001 vs. FBS + Ad-ERα/Ad-OMI.

Then, we investigated whether ERα-NRF1-OMI signaling regulates phenotype switching and proliferation of VSMCs *in vitro*. As shown in Figure 6, both ERα and OMI overexpression inhibited the upregulated expression of PCNA and OPN and the percentage of EdU-positive cells while restoring the inhibited expression of SM-MHC, α-SMA, calponin, and caldesmon by FBS. In addition, FCCP further promoted the contractile phenotype switching of VSMCs and impeded the synthetic phenotype switching and proliferation of VSMCs, while 3-MA acted in the opposite manner. Collectively, these data reveal that the ERα-NRF1-OMI signaling pathway attenuates synthetic phenotype switching and proliferation of VSMCs by restoring defective mitophagy under simulated microgravity.

### Estrogen receptor *α* regulates phenotypic switching of vascular smooth muscle cells and cerebrovascular remodeling in simulated microgravity rats

**FIGURE 7:**
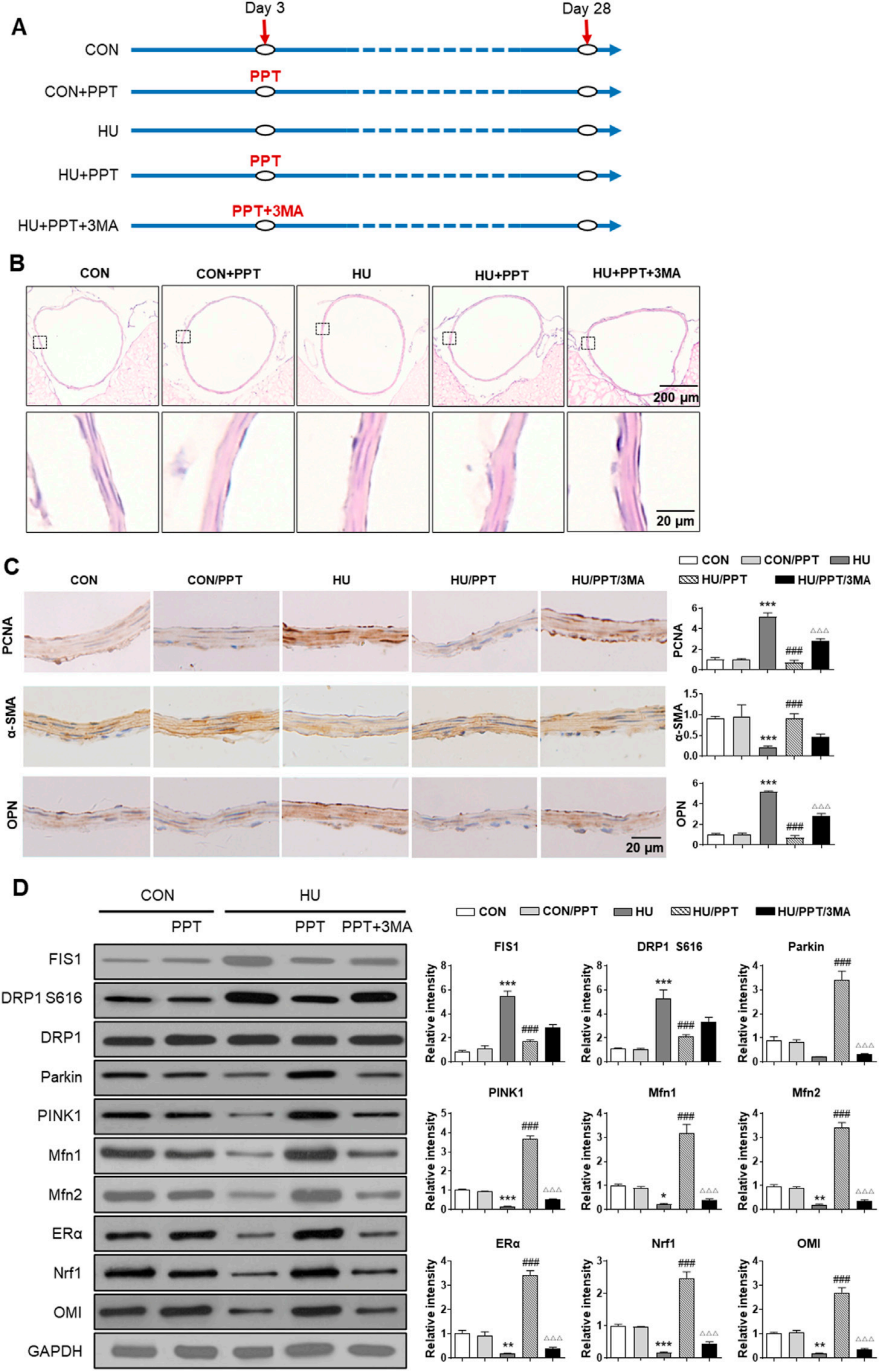
ERα regulates phenotypic switching of VSMCs and cerebrovascular remodeling in simulated microgravity rats. **(A)** Flowchart of the experimental processes and scheme of animal model establishment. **(B,C)** Representative images showing H&E staining **(B)** and immunohistochemical staining of α-SMA, OPN and PCNA **(C)** of rat basilar arteries under HU or control. **(D)** Western blot analysis of Fis1, p-Drp1 S616, Drp1, Parkin, Pink1, Mfn1/2, ERα, Nrf1, and OMI protein in rat cerebral VSMCs under HU or control. PPT was used as ERα agonist and 3 MA was used as mitophagy inhibitor. *p < 0.05, **p < 0.01 and ***p < 0.001 vs. control, ^###^ *p* < 0.001 vs. HU, ^ΔΔΔ^ *p* < 0.001 vs. HU + PPT.

Finally, we investigated the expression profiles of Fis1, Drp1, Parkin, Pink1, Mfn1/2, ERα, Nrf1 and OMI in cerebral VSMCs and whether ERα regulates cerebrovascular remodeling in HU rats. Compared with the control, HE staining showed a thickened arterial wall of HU rat cerebral arteries, which was inhibited by PPT (Figure 7B). HU-induced upregulation of OPN and PCNA and downregulation of α-SMA were restored by PPT (Figure 7C). The protein abundance of Parkin, Pink1, Mfn1/2, ERα, Nrf1 and OMI in HU rat cerebral VSMCs was significantly inhibited, while the protein abundance of Fis1, p-Drp1 S616 and Drp1 in HU rat cerebral VSMCs was significantly upregulated (Figure 7D). Subcutaneous injection of PPT partially reversed these changes, which was then impeded by 3-MA. Taken together, we revealed that ERα regulated phenotypic switching of VSMCs and cerebrovascular remodeling in HU rats by protecting mitophagy.

## Discussion

The results of this study suggest, for the first time to the best of the authors’ knowledge, that ERα regulates phenotype switching and proliferation of vascular smooth muscle cells through the NRF1-OMI-mitophagy signaling pathway under simulated microgravity.

Spaceflight is known to impose changes on human vasculature with unknown molecular etiologies. Region-specific vascular remodeling during microgravity exposure contributes to postflight cardiovascular deconditioning and orthostatic intolerance (Ren et al., 2011; Zhang et al., 2012; Peng et al., 2015; Liu et al., 2021). Ground-based microgravity simulation showed synthetic phenotypic transition and proliferation of cerebral VSMCs (Zhang et al., 2020a; Zhang et al., 2020b), and mitochondrial oxidative stress plays an important role in this process (Zhang et al., 2020a). Multiomics and systems biology analyses using biomedical profiles from fifty-nine astronauts and data from NASA’s GeneLab derived from hundreds of samples flown in space found that spaceflight causes changes in gene expression related to mitochondrial ATP synthesis, mitochondrial electron transport and oxidative phosphorylation (Da Silveira et al., 2020), which indicates that mitochondrial dysfunction is a key factor in cardiovascular deconditioning and cardiovascular diseases related to microgravity exposure. In mitochondrial dysfunction, both perturbations in proteostasis and impairment of mitochondrial metabolic functions activate the UPR^mt^. The activation of CHOP, ERα and SIRT3 has been indicated in UPR^mt^, which reduces proteotoxic stress and maintains mitochondrial integrity (Papa and Germain, 2014). However, whether ERα regulates vascular remodeling during microgravity exposure is unclear. In the present study, we found that ERα was positively associated with contractile phenotype switching but inversely correlated with synthetic phenotype switching and proliferation of VSMCs both *in vivo* and *in vitro*. Loss of ERα is correlated with reduced mtDNA copy number, disturbed mitochondrial biogenesis and respiration, and perturbed fission-fusion-mitophagy signaling. Mechanistically, our data suggested that the ERα-NRF1-OMI signaling pathway regulated Parkin stability through the UPS. In line with this, we found that activating ERα signaling by PPT could alleviate the synthetic phenotype switching and proliferation of cerebral arteries by reestablishing fission-fusion-mitophagy hemostasis. To our knowledge, we are the first to reveal that the ERα-NRF1-OMI signaling pathway participates in cerebrovascular remodeling under simulated microgravity.

ERα plays critical roles in regulating mitochondrial function, including bioenergetics, dynamics, and respiration (Klinge, 2020). Impaired mitochondrial fission-fusion flux is an important contributor to altered mitochondrial bioenergetics. Uncontrolled mitochondrial fission produces large amounts of mitochondrial debris that damage the mitochondrial genome and aberrant cristae, which disrupt normal mitochondrial respiration and ATP production (Xian and Liou, 2019). In MCF7 human breast cancer cells, E2 induced mitochondrial fission through phosphorylation of Drp1 at S616 *via* ERα (Oo et al., 2018). However, ERα depletion in MIN6 β-cells markedly reduced both mitochondrial fission and fusion proteins, including Drp1, Mff, Mfn1, Mfn2 and OPA1, and the mitochondria exhibited an elongated phenotype (Zhou et al., 2018). In the absence of ERα, there is an increase in cellular respiration and ROS production that is associated with aberrant mitochondrial cristae morphology (Zhou et al., 2018). Both the mitochondrial fission proteins Drp1, Drp1^Ser616^, and FIS1 and the mitochondrial fusion protein Mfn2 were significantly reduced in *Esr1*-KD 3T3L1 adipocytes (Zhou et al., 2020). *Esr1* knockdown induced the mitochondria toward a fusion phenotype in brown adipose tissue but not white adipose tissue (Zhou et al., 2020). In skeletal muscle, the loss of ERα significantly increased Drp1 phosphorylation at the inhibitory residue Ser637 and the expression of OPA1 and MFN2, promoting mitochondrial fusion even when the fission-related outer mitochondrial membrane Drp1 anchoring protein FIS1 was markedly reduced (Ribas et al., 2016). Besides, the myotube Esr1 deficiency caused a 50% reduction in fatty acid oxidation and a concomitant increase in fatty acid storage into complex lipids relative to control myotubes (Ribas et al., 2016). Therefore, the ERα depletion-induced mitochondria morphology is not only determined by fission and fusion proteins but also the energy metabolic state and cellular requirements. In the present study, both the HU-induced and FBS-treated synthetic transition and proliferation of VSMCs were associated with uncontrolled mitochondrial fission and inhibited mitochondrial fusion, which were reversed by ERα overexpression. These data are consistent with previous studies showing that synthetic conversion of VSMCs and hyperproliferation of pulmonary VSMCs were accompanied by mitochondrial fragmentation, decreased glucose oxidation and increased fatty acid oxidation (Bonnet et al., 2006; Salabei and Hill, 2013). In aggregate, the decreased expression of ERα seems to promote mitochondrial fission even in synthetic VSMCs, and the underlying mechanism needs to be clarified.

It has been suggested that a reduced mitochondrial membrane potential initiates mitophagy to remove damaged mitochondria and avoid excessive ROS generation. This process requires mitochondrial fission and division of damaged organelles from the network (Palikaras et al., 2018). Under stress conditions, reduced ATP production, which negatively regulates mTORC1 and ROS/BNIP3 and increases mPTP opening, can initiate mitophagy signaling. As described previously, upon exposure to microgravity, mitochondrial ROS increased mPTP opening and fission signaling, decreased membrane potential and deteriorated ATP production and respiration (Zhang et al., 2020a; Liu et al., 2021). However, mitophagy was inhibited under simulated microgravity in the present study, which disrupted the cellular self-repair mechanism of VSMCs. Furthermore, the decreased levels of Parkin and Pink and impaired mitophagy were reversed by ERα, indicating the important role of ERα in regulating mitophagy. Previously, studies have shown that ERα strongly affects early autophagic initiation and alters LC3 nuclear-cytoplasmic translocation and thereby influences sex-biased final autophagosome formation in medaka (Mohapatra et al., 2020). ERα directly interacts with NRF2, while both ERα and ERβ affect HK2 and LC3 activities (Mohapatra et al., 2020). In postmenopausal women, upregulating ERα by estrogen induces autophagy, which downregulates NLRP3 inflammasome-IL1β-mediated inflammation and pyroptosis in vascular endothelial cells (Meng et al., 2021). The loss of ERα decreased the expression of Parkin and Pink1 in Min6 β-cells, C2C12 myotubes and skeletal muscle (Ribas et al., 2016; Zhou et al., 2018), while Esr1-KD 3T3L1 adipocytes and gWAT and iWAT of female ERα knockout mice significantly increased the expression of Parkin (Zhou et al., 2020). These results suggest that mitochondrial fusion-fission-mitophagy signaling is complex, and energy demand and metabolic features should be considered when interpreting the tissue-specific alterations after ERα depletion.

The UPR^mt^-ERα axis is dependent on ERα, and its downstream protein OMI is a protease that can impede misfolded proteins in mitochondria and maintain the normal function of mitochondria. Recently, studies have shown that loss of OMI increases the number of dysfunctional mitochondria, subunits of the ETC and ROS production and decreases ATP generation (Moisoi et al., 2009). It was found that when MEFs were treated with stressors, autophagy was activated, which paralleled the increased OMI levels, suggesting that OMI expression-induced autophagy enhancement may be a protective phenomenon (Han et al., 2008). Numerous studies have discovered that animals lacking OMI/HtrA2 or with OMI/HtrA2 inhibition exhibited deficiencies in autophagy and symptoms of accelerated aging (Zhou et al., 2012; Xu et al., 2018), and overexpression of OMI/HtrA2 in cells promoted autophagy by degrading the Beclin1 inhibitory protein HAX1 (Li et al., 2010). Unlike autophagy, evidence of mitophagy regulation by OMI/HtrA2 is lacking. To determine the roles of OMI/HtrA2 and its underlying mechanism, the expression and protein stability of parkin and its role in macroautophagy and mitophagy were examined. The results showed that Parkin levels were decreased and mitophagy was inhibited after OMI downregulation, and Parkin expression and mitophagy were preserved after ERα and OMI overexpression. Further research suggested that Parkin stability is preserved in the presence of OMI, indicating that UPR–ERα-OMI signaling activates mitophagy to maintain mitochondrial homeostasis in VSMCs.

Mitophagy and mitochondrial dynamics are closely related to the phenotype of VSMCs. Previous studies have shown that uncontrolled mitochondrial fission results in ROS overproduction (Huang et al., 2016) and generates excessive mitochondrial debris that damages the mitochondrial genome and disrupts normal mitochondrial respiration and ATP production (Xian and Liou, 2019). Furthermore, mitochondrial debris promotes the opening of the mitochondrial permeability transition pore (mPTP) and the leakage of cytochrome c, which ultimately induces mitochondria-dependent apoptosis (Mao et al., 2022). In Ang II-infused mice, the dedifferentiation of VSMCs and vascular remodeling are associated with increased levels of p-DRP1 S616 (Lu et al., 2020), while Mdivi-1 inhibits fission and blocks Ang II-induced phenotypic switching of VSMCs (Deng et al., 2021). In spontaneously hypertensive or atherosclerosis-prone rats, Mfn-2 levels were diminished in VSMCs, while Mfn2 overexpression inhibited the proliferation of VSMCs and sensitized them to H_2_O_2_-induced apoptosis (Guo et al., 2007). Previously, we found increased mPTP, a reduced mitochondrial respiratory control ratio, increased fission, and inhibited fusion in HU rat cerebral VSMCs, which was reversed by MitoTEMPO (Zhang et al., 2014b; Liu et al., 2021). In the present study, we further confirmed that disturbed mitochondrial dynamics were involved in the synthetic phenotype switching of VSMCs and were associated with inhibited mitochondrial biogenesis and mitochondrial respiration. Furthermore, we also investigated the roles of ERα in mitochondrial dynamics and found that excessive mitochondrial fission was protected by ERα activation both *in vitro* and *in vivo*. However, in E2-treated MCF7 cells, phosphorylation of Drp1 at S616 is dependent on ERα (Oo et al., 2018). In our opinion, the completely different characteristics of the two cell lines may be the underlying mechanism.

The limitations still exist. Both *in vivo* and *in vitro* models were used in present work to investigate the molecular mechanism of phenotypic switching of VSMCs. We intended to investigate the molecular mechanism on cell-based models and perform phenotypic verification on animal-based models. Although the *in vivo* serum-induced A7r5 dedifferentiation cannot fully simulate the increased transmural pressure of HU rat cerebral VSMCs, the previous studies have shown that there are common mechanisms such as the T-type CaV3.1 channel and RhoA kinase in serum-induced and microgravity-induced synthetic switching of VSMCs (Wu et al., 2019; Zhang et al., 2020b).

In conclusion, the findings of the current study clarified a novel mechanism of UPR^mt^-mitophagy in which inhibited ERα-NRF1-OMI signaling and mitophagy resulted in phenotypic switching of VSMCs and cerebrovascular remodeling under simulated microgravity. Further studies are needed to elucidate the mechanism of activation of the ERα axis of the UPR^mt^ during microgravity exposure. Additionally, further research is needed to examine the interaction between the ERα and SIRT3 axes of UPR^mt^ and their roles in vascular remodeling. A full understanding of the ERα axis in UPR^mt^ enables targeting of the mitochondrial network for intervention in vascular remodeling and postflight cardiovascular deconditioning in astronauts.

## Data Availability

The raw data supporting the conclusion of this article will be made available by the authors, without undue reservation.
