# Supplementary material for: Estrogen receptor α regulates phenotypic switching and proliferation of vascular smooth muscle cells through the NRF1-OMI-mitophagy signaling pathway under simulated microgravity
Source: Front Physiol. 2022 Nov 10;13:1039913. doi: 10.3389/fphys.2022.1039913 (PMC9691402; doi:10.3389/fphys.2022.1039913)
Supplement: Supplementary file 1 [file Presentation1.pptx]

## Slide 1
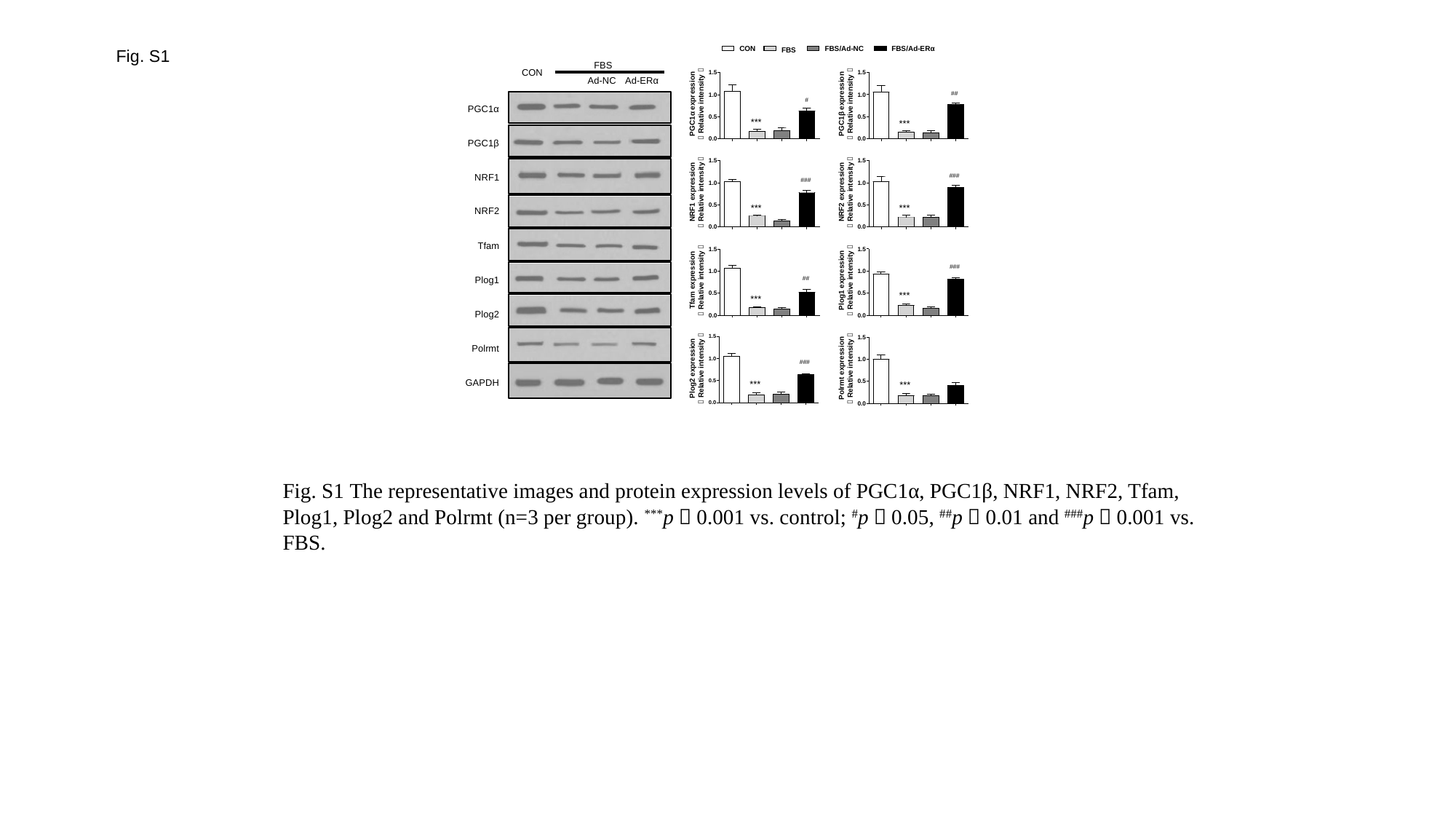

FBS/Ad-NC
FBS/Ad-ERα
CON
Fig. S1
FBS
 FBS
CON
Ad-NC
Ad-ERα
PGC1α
PGC1β
NRF1
NRF2
Tfam
Plog1
Plog2
Polrmt
GAPDH
##
PGC1α expression
（Relative intensity）
PGC1β expression
（Relative intensity）
#
***
***
###
###
NRF1 expression
（Relative intensity）
NRF2 expression
（Relative intensity）
***
***
###
Tfam expression
（Relative intensity）
Plog1 expression
（Relative intensity）
##
***
***
###
Plog2 expression
（Relative intensity）
Polrmt expression
（Relative intensity）
***
***
Fig. S1 The representative images and protein expression levels of PGC1α, PGC1β, NRF1, NRF2, Tfam, Plog1, Plog2 and Polrmt (n=3 per group). ***p＜0.001 vs. control; #p＜0.05, ##p＜0.01 and ###p＜0.001 vs. FBS.
